# A sub-150-nanometre-thick and ultraconformable solution-processed all-organic transistor

**DOI:** 10.1038/s41467-021-26120-2

**Published:** 2021-10-06

**Authors:** Fabrizio Antonio Viola, Jonathan Barsotti, Filippo Melloni, Guglielmo Lanzani, Yun-Hi Kim, Virgilio Mattoli, Mario Caironi

**Affiliations:** 1 Center for Nano Science and Technology @PoliMi, Istituto Italiano di Tecnologia, via Pascoli 70/3, 20133 Milano, Italy; 2 Dipartimento di Fisica, Politecnico di Milano, Piazza Leonardo da Vinci 32, 20133 Milano, Italy; 3 Department of Chemistry & ERI, Gyeongsang National University, Jin-ju, 660-701 Republic of Korea; 4 Center for Materials Interfaces, Istituto Italiano di Tecnologia, viale Rinaldo Piaggio 34, 50125 Pontedera, PI Italy

**Keywords:** Electrical and electronic engineering, Electronic devices, Electronic devices

## Abstract

Recent advancements in the field of electronics have paved the way to the development of new applications, such as tattoo electronics, where the employment of ultraconformable devices is required, typically achievable with a significant reduction in their total thickness. Organic materials can be considered enablers, owing to the possibility of depositing films with thicknesses at the nanometric scale, even from solution. However, available processes do not allow obtaining devices with thicknesses below hundreds of nanometres, thus setting a limit. Here, we show an all-organic field effect transistor that is less than 150 nm thick and that is fabricated through a fully solution-based approach. Such unprecedented thickness permits the device to conformally adhere onto nonplanar surfaces, such as human skin, and to be bent to a radius lower than 1 μm, thereby overcoming another limitation for field-effect transistors and representing a fundamental advancement in the field of ultrathin and tattoo electronics.

## Abstract (web summary)

Reducing the thickness of flexible electronics is crucial to achieve better conformability and mechanical flexibility. Here, the authors demonstrate all-solution processed OFET with total thickness < 150 nm, with high transparency, which allows the device to conformally adhere onto non-planar surfaces, such as human skin.

## Introduction

Continuous advancements in the field of ultraflexible electronics and in reducing device thickness have boosted the development of conformable electronic components, serving various applications, spanning from wearable electronics^1^ to so-called “tattoo electronics” or “skin-laminated electronics”^2–4^. Conformable electronics refers to a class of devices with the ability to adhere onto complex shaped surfaces, adapting their morphology to local details of the target surface (e.g., local roughness)^5^. This ability strictly relies on both the intrinsic mechanical properties of the adopted materials and the device architecture.

In general, both the conformability and mechanical flexibility of a specific material are determined by its bending stiffness: on the one hand, they can be improved by reducing the Young’s modulus of the material^6^. On the other hand, since the bending stiffness has a cubic dependence on the material thickness^7,8^, another typical way to improve conformability and flexibility is to reduce the total thickness. For this reason, most of the reported approaches in the literature for the fabrication of ultraflexible and ultraconformable electronics are based on superthin (few micrometres thick) or ultrathin structures (thickness below 1 μm), according to the definition reported by Nawrocki^9^.

In this emerging field of conformable devices, organic electronics is one of the most exploited options thanks to the peculiar and intrinsic characteristics of the employed materials, typically hardly achievable with inorganic counterparts^10,11^. Indeed, organic materials permit the achievement of ultrathin functional nanosheets that are intrinsically soft, flexible and transparent. Moreover, since they can be made solution processable, they are compatible with several large areas and cost-efficient deposition techniques, such as printing^12–15^, and require low processing temperatures^16–18^.

Since the substrate typically represents the thickest layer in an electronic device stack, its scaling has mainly driven progress in this context. To date, Parylene C is probably the most employed material as a substrate for the fabrication of superthin and ultrathin devices due to its mechanical properties and the possibility of being deposited through chemical vapour deposition (CVD), controlling its thickness with high precision, down to a few tens of nanometres. Bonfiglio and collaborators reported the first example of organic ultrathin devices, adopting a Parylene C nanosheet as the substrate and gate dielectric to produce freestanding organic field-effect transistors (OFETs)^19^. Afterwards, analogue structures have been reported for a single-stage amplifier^20^, multimodal sensor^21^ and printed logic circuits^22–25^.

The works of Takao Someya’s group pushed even further the thickness limit for ultrathin organic transistors. They presented a 270-nm-thick OFET fabricated over Parylene diX-SR, employed as a touch sensor directly onto human skin, able to reach an impressive bending radius of 1.5 μm^26^.

In addition to the use of Parylene C, alternative insulators have been exploited for superthin and ultrathin organic transistors. For instance, freestanding OFETs fabricated over poly(vinyl alcohol) (PVA)^27–30^ and poly-acrylonitrile (PAN) nanosheets have been demonstrated^31,32^.

In all the reported works so far^19–32^, the fabrication process requires at least one step in high-vacuum conditions (such as thermal evaporation or CVD). To date, there is no demonstration of ultrathin transistors in which each part of the stack (i.e., substrate, electrodes, semiconductor, gate dielectric) is fully solution processed. The latter approach would greatly simplify fabrication process flows, enabling easier upscaling at reduced costs.

Here, we demonstrate that it is possible to fabricate an all solution-processed OFET with a total thickness <150 nm, which is the thinnest freestanding transistor ever fabricated, by adopting an approach based on solution-assisted delamination of freestanding ultrathin insulating poly(vinyl formal) (PVF) layers employed as a self-standing (or self-supporting) gate dielectric. As a consequence, we also demonstrate the smallest bending radius (0.7 µm) reported thus far for any transistor technology. Due to its ultralow thickness, the device shows high transparency, together with an extremely high level of conformability, and is able to conform on complex 3D surfaces, such as human skin. Such a result pushes even further the boundaries of ultrathin organic electronics towards solution-based and large-area produced imperceptible systems suitable for integration on top of prefabricated objects to make them “smarter” or “connected” without altering their aspect, with simple lamination processes driven by van der Waals forces. In addition to higher mechanical robustness and flexibility, a thinner device implies lower volumes of materials, especially substrates, a critical aspect for the sustainability of electronics conceived to be disposable.

## Results

**Fig. 1:**
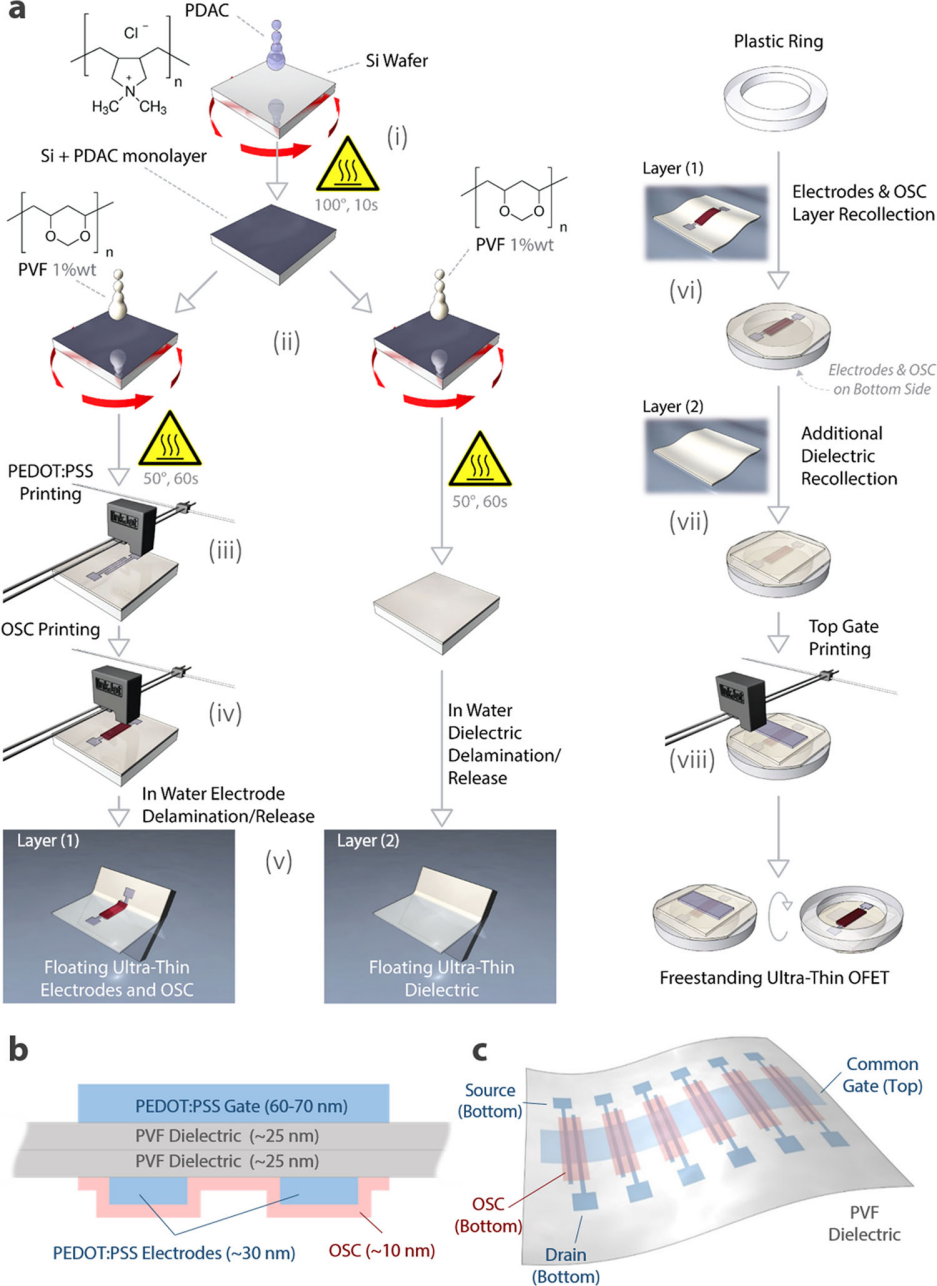
Schematic representation of the fabrication process and of the structure of ultraconformable solution-processed transistors. **a** Fabrication process flow for all-organic ultrathin OFETs: (i) PDAC spin-coating, (ii) layer-1 and layer-2 PVF spin-coating, (iii) ink-jet printing of PEDOT:PSS source/drain electrodes on layer-1, (iv) ink-jet printing of the organic semiconductor onto the electrodes over layer-1, (v) layer-1 and layer 2 delamination in water, (vi) layer-1 recollection on a plastic ring, (vii) recollection of layer-2 on layer-1, (viii), ink-jet printing of PEDOT:PSS gate electrodes. **b** Lateral view scheme and **c** overview of the ultrathin all-organic transistors.

PVF nanosheet fabrication relies on the adaptation of the process originally proposed by Baxamusa’s group (see the “Methods” section for details)^33^. We use a functionalized silicon wafer over which an ultrathin PVF nanosheet is deposited by spin-coating. The functionalization is based on a subnanometric layer of a poly(diallyldimethylammonium chloride) (PDAC) that strongly adheres to the silicon wafer, establishing a favourable free energy condition that promotes spontaneous delamination of the PVF nanosheet once the wafer is placed in contact with water. Once delaminated, the nanosheet remains well spread out, floating on the water surface due to its strong hydrophobic behaviour. As such, it provides a very flat, homogeneous and robust freestanding dielectric layer. With respect to the dry delamination (or peeling) procedure, the liquid-mediated delamination (or wet delamination) process guarantees lower stresses in the film, and in additional layers eventually deposited on top, since, under the right conditions, it is thermodynamically favoured^33^. We report the device fabrication strategy in Fig. 1a and the architecture of the realized OFETs in Fig. 1b, c. The latter layout is the result of the coupling of two different ultrathin PVF nanosheets using a multistep fabrication procedure. We refer to them as layer-1 and layer-2, respectively. As the first step, we deposited layer-1 on the silicon wafer (Fig. 1a (i-ii)), and we patterned source and drain electrodes on it by ink-jet printing poly(3,4-ethylenedioxithiophene):polystyrene sulfonate (PEDOT:PSS, Fig. 1a (iii)). These electrodes show a very high level of transparency. As a consequence, connection with external probes for electrical testing results is challenging. To overcome this issue, contact pads were patterned by ink-jet printing a silver nanoparticle-based ink at the extremes of the PEDOT:PSS electrodes. Such electrodes are clearly visible and facilitate the electrical characterization of the transistors. However, they are not required for the correct operation of any transistor presented here. For the organic semiconductor (OSC), we explored different donor-acceptor copolymers, which are representative of a relevant class of solution-processable and printable semiconducting materials, which we patterned onto the electrodes by inkjet printing as well (Fig. 1a (iv)). In particular, we employed both a (poly[2,5-bis(7-decylnonadecyl)pyrrolo[3,4-c]pyrrole-1,4(2H,5H)-dione-(E)-1,2-di(2,2′-bithiophen-5-yl)ethene] (29-DPP-TVT) and 3,6-diketopyrrolopyrrole-alt-5,5-(2,5-di(thien-2-yl)thieno [3,2-b]thiophene) DPP-TTT (data reported in Supplementary Figs. 3–4). In parallel, we prepared layer 2 on another silicon wafer (Fig. 1a (v)). We then proceeded to couple the two layers. In detail, we delaminated layer-1, obtaining a floating freestanding nanosheet with ink-jet printed electrodes and a semiconductor facing upward (see Supplementary Video 1). Afterwards, we recollected floating layer-1 using a plastic ring approaching the nanosheet from the top (Fig. 1a (vi)). At this point, we delaminated the pristine PVF nanosheet (layer-2), recollecting it with the plastic ring carrying freestanding layer-1 (Fig. 1a (vii)). As a result, layer-2 is stacked on top of the dielectric side of layer-1, forming the double-layer gate dielectric of the freestanding OFET. To complete the device, we ink-jet printed the PEDOT:PSS gate electrode directly on top of the freestanding system (Fig. 1a (viii)). It is important to emphasize that we adopted a PVF bilayer as the dielectric to limit the possibility of short circuits from the top printed gate to the bottom layers since single PVF nanosheets could sporadically display microscopic pinholes. By adopting this strategy, the probability of observing a short circuit is drastically reduced, since it is very unlikely for two pinholes of different layers to overlap when two nanosheets are recollected one on top of the other^34^.

**Fig. 2:**
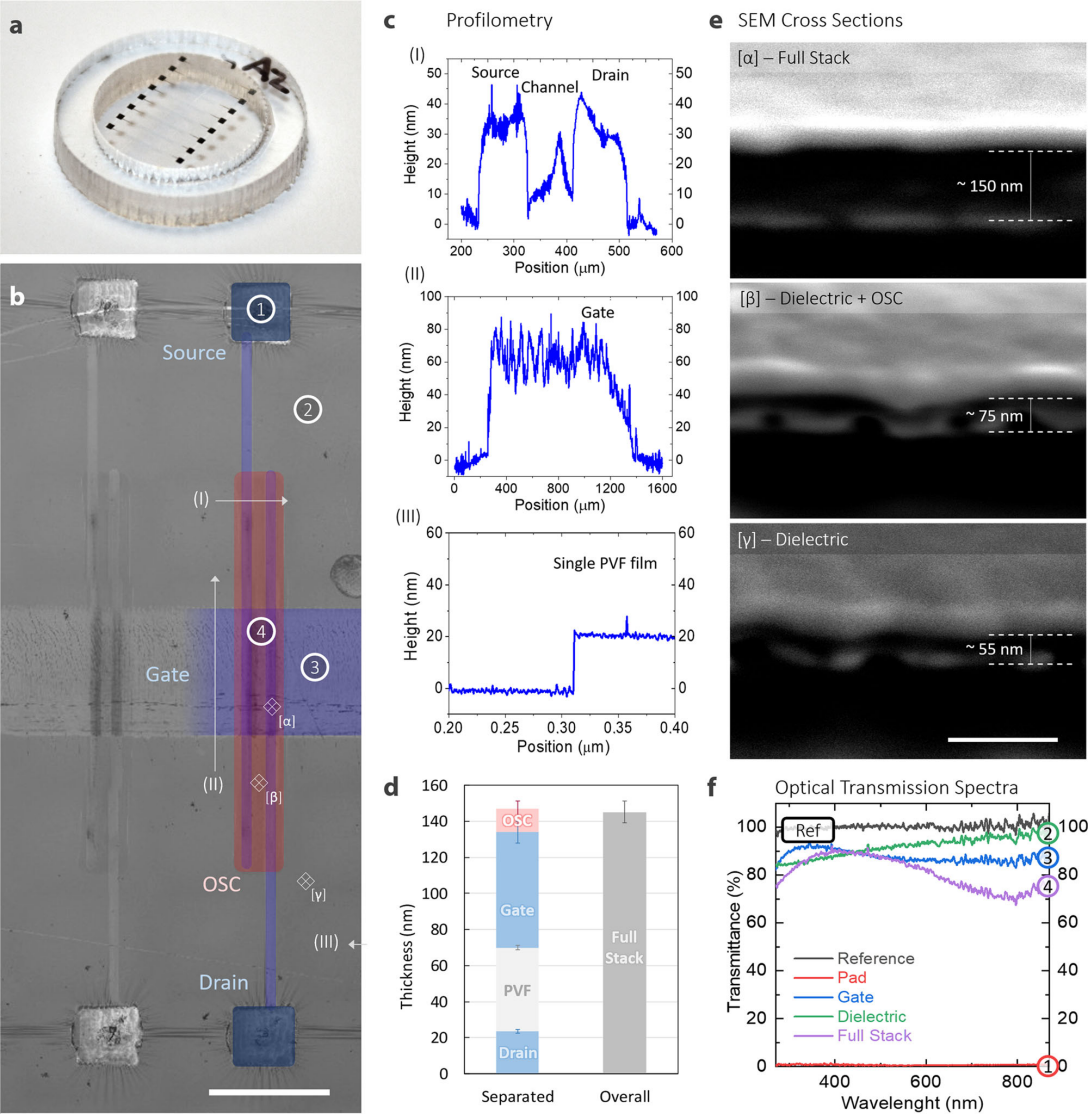
Thicknesses and optical transmission spectra. **a** Photograph of the final freestanding ultrathin OFET arrays suspended on a plastic ring. **b** Optical microscope image of two freestanding devices (right one colorized for clarity, scale bar 1 mm). Profilometry locations, scanning electron microscopy cross sections and optical spectral measurements (**c**, **e**, **f**) are highlighted by numbered arrows, diamonds and circles, respectively. **c** Profile measurements of the device in different locations: (I) source-drain channel and organic semiconductor; (II) gate electrode; (III) single PVF dielectric film. **d** Results of thickness measurement (mean and standard deviation) by profilometry of the different layers and of the overall device in a statistically significant number of samples (10 measurements for the full stack, 10 measurements for the PVF layer, 8 for the source/drain electrodes, 8 for the OSC, 6 for the gate electrode). **e** Scanning electron microscopy images of the cross section of the device in different positions obtained by focus ion beam etching (sample tilt 52°, scale bar 200 nm): section of the full stack (α), section of dielectric and organic semiconductor (β) and section of only dielectric (γ). **f** Optical transmission spectra measured in different positions (measurement spot diameter ~300 µm): (1, red line) Ag covered pad transmittance, (2, green line) dielectric layer transmittance, (3, blue line) dielectric layer over bottom gate transmittance, (4, purple line) full stack transmittance; black line is lamp reference signal.

We show a photograph of the assembled freestanding OFET in Fig. 2a, while its optical microscopy picture is reported in Fig. 2b, where colorized areas highlight the different functional parts (source, drain, gate and OSC).

We performed profilometry measurements of each single layer forming the structure (directly on the silicon wafer before delamination) and of the full stack device (after delamination from the wafer). The thickness of a single PVF film layer is ~25 nm, producing an ~50 nm thick dielectric bilayer. The thickness of the bottom gate is less than 70 nm and that of the top source/drain electrodes covered by the semiconductor is ~40 nm, as shown in Fig. 2c. In Fig. 2d, we compare the total thickness statistics obtained both by measuring the full stack and by summing the thickness obtained for each single layer in the stack. The two values are in very good agreement, returning a total device thickness of 143 ± 7 nm, thus qualifying the proposed OFET as the thinnest ever produced. Scanning electron microscopy (SEM) images of the device cross section obtained by means of focused ion beam (FIB) milling further support the thickness analysis (Fig. 2e). The extreme thinness of PEDOT:PSS electrodes and of the semiconductor results in a high transparency of the device, with an overall average transmittance of more than 80% in the visible range for the full stack (see Fig. 2f for the transmittance spectra of the device). The transmittance value can reach 90% considering the weighted average obtained by the transmittance spectra of the different elements of the device and their coverage area, as reported and explained in the Experimental Section. Such a level of transparency exceeds the values obtained with other approaches adopted thus far for ultrathin devices^19–21,23–32^.

**Fig. 3:**
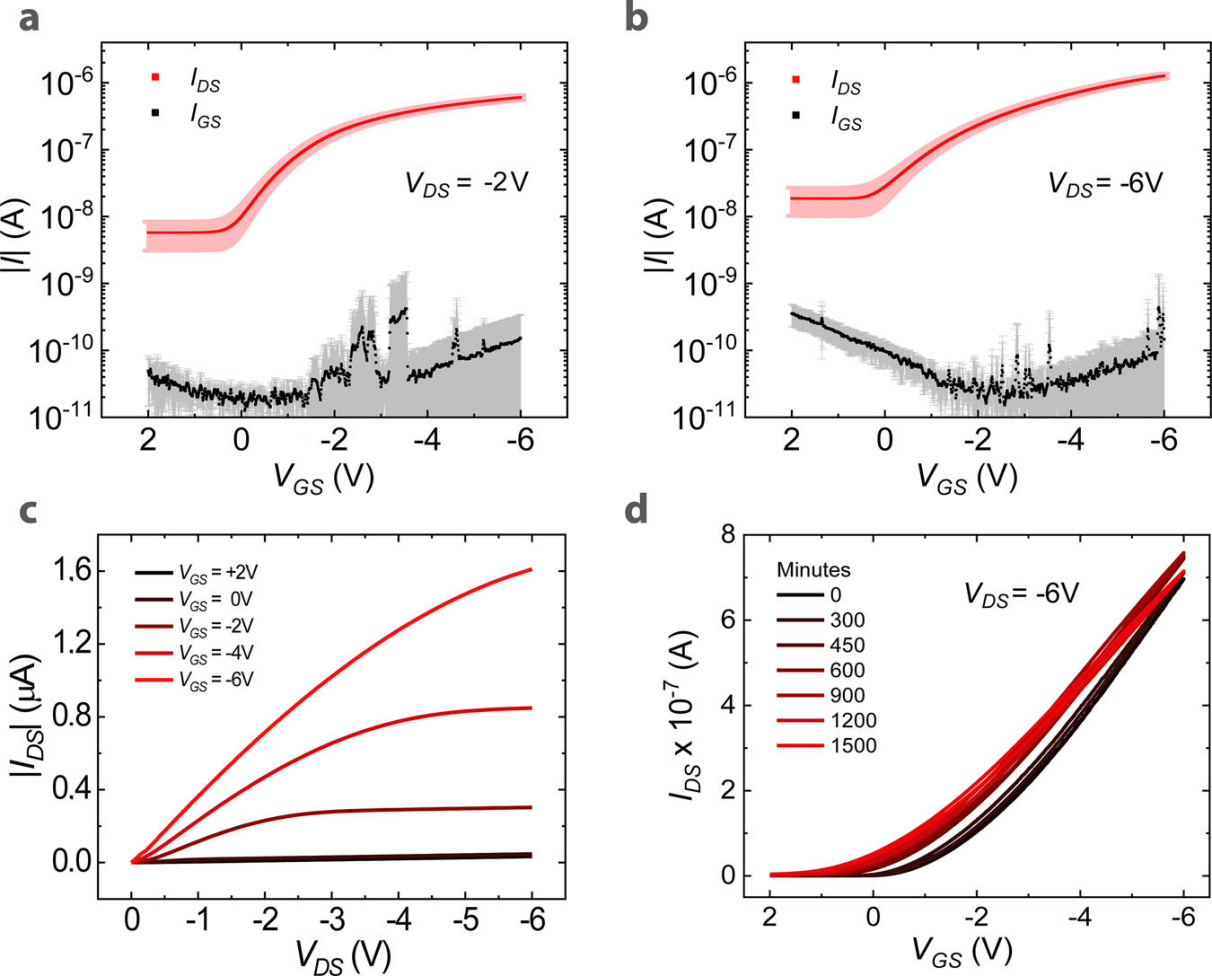
Electrical characterization of the organic transistors. Average transfer characteristic curves, with their standard deviation, obtained on eight ultrathin devices based on 29-DPP-TVT in **a** linear and **b** saturation regimes. **c** Typical output characteristic curve for a 29-DPP-TVT-based ultrathin transistor. **d** Transfer characteristic curve acquired consecutively in ambient air as a function of time for an ultrathin transistor.

The electrical characterization of the freestanding OFETs is presented in Fig. 3, where we report the data averaged for an array of eight devices. Thanks to the scaling of the gate dielectric thickness, its areal capacitance approaches the value of 60 nF/cm^2^ (see Supplementary Fig. 5 for the complete impedance measurements), thus allowing the low-voltage operation of the devices in both the linear and saturation regimes, with an average threshold voltage of 0.9 ± 0.2 V in saturation. As appreciable from the average transfer curves with their standard deviation (Fig. 3a–b), the devices have very good reproducibility over the array (see Supplementary Fig. 4 for the raw data). We also verified good run-to-run reproducibility (see Supplementary Fig. 11). Despite the ultrathin dielectric layer, for all the presented transistors, the *I*_GS_ gate leakage currents are significantly low, with values below 700 pA in the linear regime and below 1 nA at saturation. Furthermore, from the shape of the output characteristic curve (Fig. 3c), it is possible to appreciate the proper operation of the transistor.

We extracted the apparent field‐effect mobility (*µ*) according to the gradual channel approximation. The average value is 0.092 cm^2^ V^−1^ s^−1^ in the linear regime (with *V*_DS_ = −2 V) and 0.098 cm^2^ V^−1^ s^−1^ in the saturation regime (with *V*_DS_ = −6 V) with a *V*_GS_ (gate voltage) dependence, as shown in Supplementary Fig. 6. We then calculated the effective mobility (*µ*_eff_) through the formula: *µ*_eff_ = *µ* × *r*, where *r* is the reliability factor, as described by a recent paper by Choi et al.^35^. Since we obtained high average values for *r*, reaching 91% in the linear regime and 88% in the saturation regime, the effective mobilities (Supplementary Table 1) were very close to the apparent *µ* values (Supplementary Table 3; extraction of *R*_C_ is reported in Supplementary Table 4). Such solid estimation indicates that mobility achieved with ultrathin devices is in line with what has been consistently reported for conventional low-voltage, printed, DPP-TVT-based OFETs^36^.

Regarding the environmental stability of the devices, which is a fundamental requirement for applications such as wearable and tattoo electronics, we performed a proof-of-principle test by acquiring a transfer curve of a device every 5 min for 25 h while storing it in air under environmental conditions. Such bias conditions represent the typical operation for sensor applications, where the OFET is switched on and off instead of keeping it at a constant and continuous “on” operational state, while the duration of the stability measurement was chosen to simulate a single‐day disposable usage^7^. It is worth noting that we did not introduce any further encapsulation, and we tested the self-standing OFET in air as a result of the process described above. After 25 h, the device was still operational, as shown in Fig. 3d, denoting only a concurrent increase in both the On and Off currents, which we attribute to mild p-type doping of the OSC due to its direct exposure to oxygen and moisture^37^.

**Fig. 4:**
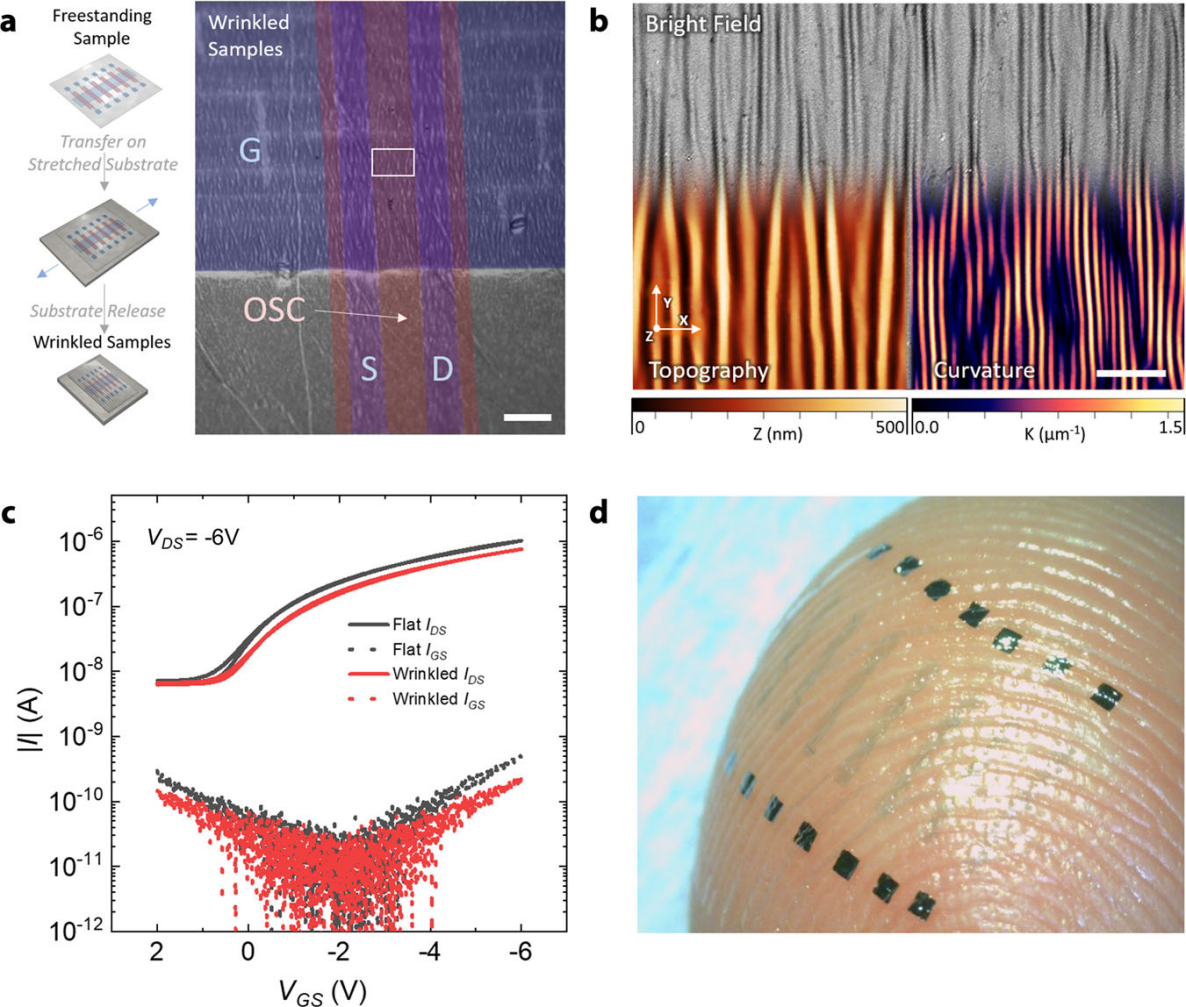
Mechanical flexibility and conformability. **a** Schematic procedure to induce wrinkling in the ultrathin transistor sample (left) and optical picture of the wrinkled transistor device (right, scale bar 100 µm); the white box indicates the point in which a specific curvature analysis is performed as reported in (**b**). **b** Merged optical image, topographic and curvature mapping of a wrinkled sample acquired in the active area of the transistor by optical profilometry at ×150 magnification (scale bar 10 µm); the curvature (K) mapping is calculated by optical profilometry data (details in Supplementary Figs. 8–9). **c** Transfer characteristic curves for an ultrathin device in saturation mode when recollected on a prestretched PDMS substrate (flat sample, black line) and after PMDS relaxation, which induces strong buckling on the surface (wrinkled sample, red lines). **d** A film sample containing an array of devices adhering to the skin to demonstrate conformal adhesion and a high level of transparency.

To investigate the conformability and mechanical flexibility of the devices, we laminated OFET arrays over a prestretched soft polydimethylsiloxane (PDMS) film with a thickness of ~3 mm, as shown in Fig. 4a (for details, see the Methods – Characterization section). The PDMS release induces wrinkles onto the devices (Fig. 4a–b) as a result of the stress at the transistor-elastomer interface originating from the mismatch between the PVF and PDMS Young’s moduli^34^. The curvature profile for the active area shows that more than 50% of the curvature maxima have a radius <1  μm, while more than 10% have a radius <0.8 μm, with a minimum of 0.7 μm achieved in several spots of the imaged area, as reported in Supplementary Figs. 8–9. We report in Fig. 4c a comparison of the transfer characteristics of a single device before and after PDMS release, while in Supplementary Fig. 12, the statistics for an array of eight devices are reported. Despite sustaining an extremely low bending radius of 0.7 μm, the transistors retain proper operation, and their electrical performance is not significantly affected by the extreme bending condition, thus reducing the previous record by a factor of 2 in the minimum bending radius^26^. Moreover, conformal adhesion on a nonplanar surface, such as human skin (Fig. 4d), is obtained and demonstrated as a result of reduced bending stiffness^38^.

**Fig. 5:**
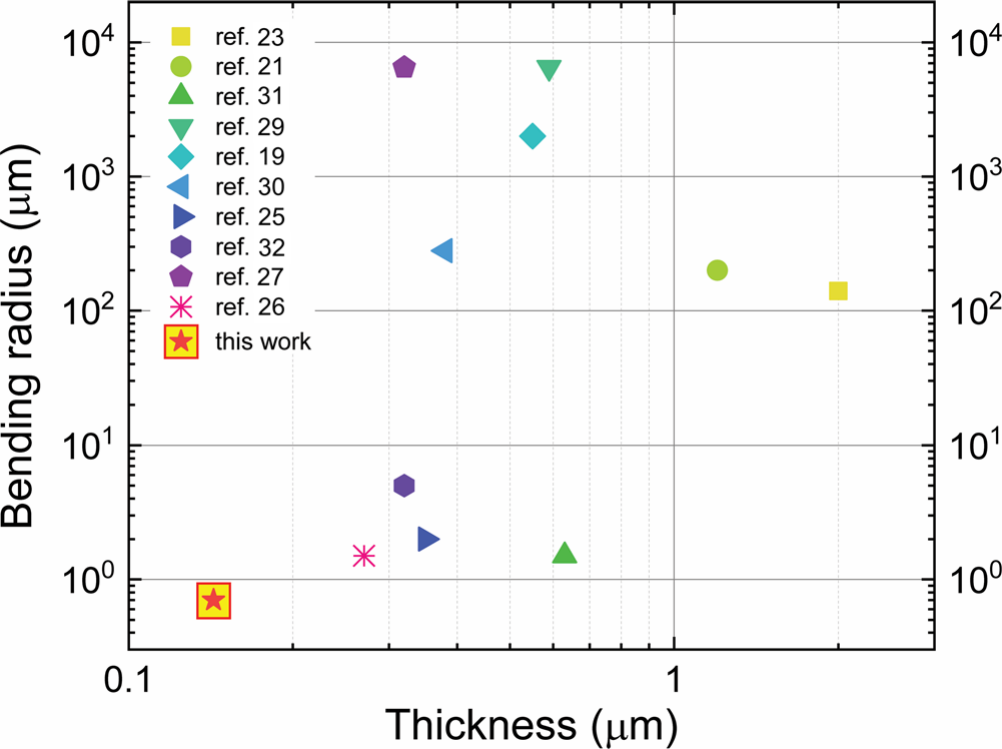
State of the art on super/ultra-thin transistors. Comparison graph of superthin and ultrathin organic field-effect transistors. In the *x*-axes, the total thickness is reported, and the minimum bending radius is in the *y*-axes.

In conclusion, we have successfully fabricated ultraflexible, sub-150-nm-thick, all-organic field-effect transistors based on an ultrathin solution-processable dielectric bilayer, adopting only solution-based techniques, such as spin-coating and inkjet printing. The devices can operate at low voltage and, thanks to their very low thickness, can conform to human skin in an imperceptible way and sustain bending radii as low as 0.7 μm, without significant variations in their electrical performance, thus setting a new limit in thickness and conformability. Moreover, as shown in Fig. 5 and Supplementary Table 4, in which we summarized the state-of-the-art ultrathin field-effect transistors, this is the first demonstration of ultrathin (<1 μm) fully solution-processable transistors, including the substrate. The electrical characterization showed very good reproducibility, demonstrating the solidity of the proposed fabrication process, and the stability test in air makes the devices in principle compatible with single-day, disposable usage. Furthermore, the employed materials and their very low thickness guarantee a high transparency. In addition to providing an alternative and valid technological solution for the fabrication of ultrathin devices, the proposed approach can be a candidate for a plethora of possible uses ranging from wearable to tattoo/epidermal electronics for sensing, health care or biomedical applications where conformability, ultraflexibility and transparency are necessary.

## Methods

### PVF nanosheets preparation

Poly(vinyl formal) (PVF) was purchased from SPI Supplies (trade name Vinylec E Polyvinyl Formal Resin). Ethyl lactate (EL, ≥ 98%, Food Chemical Codex, Food Grade) and poly(diallyl dimethylammonium chloride) (PDAC, 20% wt in water solution) were purchased from Sigma–Aldrich. Si wafers (3 inches diameter and 381 ± 25 µm thick) used for PVF nanosheet fabrication were purchased from Silicon Materials (Si-Mat). Commercial poly(methyl methacrylate) (PMMA)—Clear Astariglas® Acrylic sheet (made from 100% virgin MMA (methyl methacrylate monomer), light transmission 92%, thermal expansion 2.1 mm/m)—was purchased as 0.3 cm × 40 cm × 55 cm slabs and cut into rings with internal and external diameter of 12 mm and 18 mm, respectively. A PDAC solution (0.5% wt) was prepared by diluting the commercial 20% wt water solution with deionized water. It was stirred for 15 min immediately before its use. PVF solution (1% wt) was prepared by dissolving PVF in EL by stirring at 650 rpm and at 50 °C overnight. Immediately before use, the PVF solution was heated using a water bath at 50 °C and stirred at 650 rpm to prevent potential polymer aggregate formation. The silicon wafer surface was washed with acetone as a first step and then with isopropanol. Thereafter, the wafer was exposed to O_2_ plasma for 120 s (O_2_ pressure 0.4 mbar, power 100 W, Plasma Asher model Femto, Diener Electronic) to improve its wettability. Immediately after, the wafer was functionalized with a subnanometric PDAC layer by spin coating ~2 ml of PDAC solution at 4000 rpm for 15 s, followed by soft baking of the film at 100 °C for 10 s on a hotplate. PDAC excess was rinsed with deionized water. PVF solution was spin-coated on the PDAC layer in a two-step process for a total duration of 10 s: the first 5 s at 300 rpm and the last 5 s at 3000 rpm. Approximately 2 mL of solution was deposited for each PVF nanosheet spinning session. The PVF nanosheet was then baked for 60 s at 50 °C on a hotplate to remove the excess solvent.

### Ultra-thin OFETs

Bottom contact OFETs were fabricated employing PEDOT:PSS (Clevios PJ700 formulation, purchased from Heraeus) as source, drain and gate contacts patterned by means of ink-jet printing using a Fujifilm Dimatix DMP2831 through a cartridge with 10 pL nozzles. The source and drain were printed at a drop spacing of 45 μm (one layer), a firing voltage of 40 V, a jetting frequency of 1 kHz and a printer plate temperature of 28 °C. The gate electrode was printed at a drop spacing of 40 μm (one layer), a firing voltage of 40 V, a jetting frequency of 1 kHz and a printer plate temperature of 28 °C. No annealing of the electrodes was performed. The channel width was set to 1 mm, while the channel length was 100 μm. The total area of each transistor is 1 × 10^−3^ mm^2^.

As reported in Fig. 2d, these devices show a very high level of transparency, thus making it difficult to identify source and drain contacts without the help of visible elements. For this reason, contact pads were patterned by ink-jet printing a silver nanoparticle-based ink (Silverjet DGP-40LT-15C, purchased from Advanced Nano Products) with the scope of providing a visible element and facilitating the electrical characterization of the transistors. The area of each pad is 440 × 440 μm^2^. Silver pads are quite far (~1.2 mm) from the active area of the devices and are not required for the correct operation of any transistors presented here.

Poly[2,5-bis(7-decylnonadecyl)pyrrolo[3,4-c]pyrrole-1,4(2H,5H)-dione-(E)-1,2-di(2,2′-bithiophen-5-yl)ethene] was synthesized according to Yu et al.^39^ while 3,6-diketopyrrolopyrrole-alt-5,5-(2,5-di(thien-2-yl)thieno [3,2-b]thiophene) was synthesized using the method explained by Chen et al.^40^. They have been used as p-type OSCs patterned by means of inkjet printing using a Fujifilm Dimatix DMP2831. Both semiconductors were printed from a 1,2-dichlorobenzene-based solution at a concentration of 2.5 mg ml^−1^ through a cartridge with 10 pL nozzles. OSCs were printed at a drop spacing of 50 μm (one layer), a firing voltage of 35 V, a jetting frequency of 1 kHz and a printer plate temperature of 28 °C. No annealing of the OSCs was performed.

### Freestanding ultra-thin OFET assembly

First, an array composed of eight source/drain electrodes and the OSC filling the channel was ink-jet printed over a PVF nanosheet (layer-1) before delamination from the silicon wafer. Using a surgery blade, we cut squares of layer-1 and a second PVF nanosheet (layer-2) with suitable dimensions to fit the PMMA ring used for the recollection (internal and diameter 12 mm, external diameter 18 mm). Layer-1 and layer-2 were recollected on top of each other (see Supplementary Video 1). A common gate electrode was ink-jet printed to simultaneously print the gate for all 8 devices.

### Characterization

We performed electrical characterization (transfer curves and output curves) of the transistors in an air atmosphere using an Agilent B1500A semiconductor parameter analyser.

PVF nanosheet thickness characterization was performed by means of a mechanical profilometer (Alpha Step-IQ, KLA-Tencor).

Optical microscopic images were acquired by means of a Leica DCM 3D confocal profilometer at ×10 magnification, with multiple image stitching options. The transmission spectra average was obtained by the weighted transmission of different areas of each element of one device (transmission spectra × coverage area), as follows:

- [PVF] 92% transmission spectra × 72% coverage area;
- [PEDOT:PSS + PVF] 87% transmission spectra × 15.5% coverage area;
- [full stack] 82.5 transmission spectra × 12.5% coverage area.

For each curve, averaging was performed in the range of 400–750 nm.

FIM-milled cross-sections and SEM imaging of the ultrathin organic transistor were obtained with a Dual Beam FIB/SEM Helios Nano-Lab 600i (FEI). SEM images of cross-sections were obtained under a sample tilt angle of 52° (accelerating voltage 10 kV).

PVF nanosheet capacitance was extracted using an Agilent 4294 A Impedance Analyzer with a two-probe configuration. The samples were tested in a frequency range from 40 Hz to 1 MHz, applying a signal with a 500 mV amplitude. The capacitors were fabricated by using a couple of 25-nm-thick layers of PFV joined together. The resulting 50-nm-thick PVF layer was sandwiched between two PEDOT:PSS electrodes printed on the outer part of the PVF layer. The parallel plates have an overlapping area of 0.58 mm^2^.

The mechanical tests were performed by laminating the array of transistors over a prestretched PDMS, which was stretched at 5% before transferring the devices on it. To provide uniform extension, we used metallic clamps. The clamps were 3 cm in width, larger than the PDMS slab, which has a width of ~2 cm. The slab was then fixed at the corresponding 5% extension point using Kapton tape.

The study was performed following the principles outlined in the Helsinki Declaration of 1975, later revised in 2000. All participants signed an informed consent form before the experimental activity began after being informed about the aims and procedures of the experiments.

## Supplementary information

Supplementary Information

Description of Additional Supplementary Files

Supplementary Movie 1

## Data Availability

The data that support the findings of this study are available from the corresponding author upon reasonable request.
