# Supplementary material for: A sub-150-nanometre-thick and ultraconformable solution-processed all-organic transistor
Source: Nat Commun. 2021 Oct 6;12:5842. doi: 10.1038/s41467-021-26120-2 (PMC8494881; doi:10.1038/s41467-021-26120-2)
Supplement: Supplementary file 1 — Supplementary Information [file 41467_2021_26120_MOESM1_ESM.pdf]

# Supplementary Information

## A sub-150-nanometre-thick and ultraconformable solution-processed all-organic transistor

*Fabrizio Antonio Viola[1]*, Jonathan Barsotti[1], Filippo Melloni[1], Guglielmo Lanzani[1,2], Yun-Hi Kim[3], Virgilio Mattoli[4]* and Mario Caironi[1]**

[1] Center for Nano Science and Technology @PoliMi, Istituto Italiano di Tecnologia, via Pascoli 70/3, 20133 Milano, Italy.
E-mail: fabrizio.viola@iit.it; mario.caironi@iit.it

[2] Dipartimento di Fisica, Politecnico di Milano, Piazza Leonardo da Vinci 32, 20133 Milano, Italy

[3] Department of Chemistry & ERI, Gyeongsang National University, Jin-ju, 660-701, Republic of Korea

[4] Center for Materials Interfaces, Istituto Italiano di Tecnologia, viale Rinaldo Piaggio 34, 50125 Pontedera (PI), Italy
E-mail: virgilio.mattoli@iit.it

* Corresponding authors

Keywords: ultraconformable electronics; ultraflexible electronics; solution-processed transistors; wearable electronics; tattoo electronics

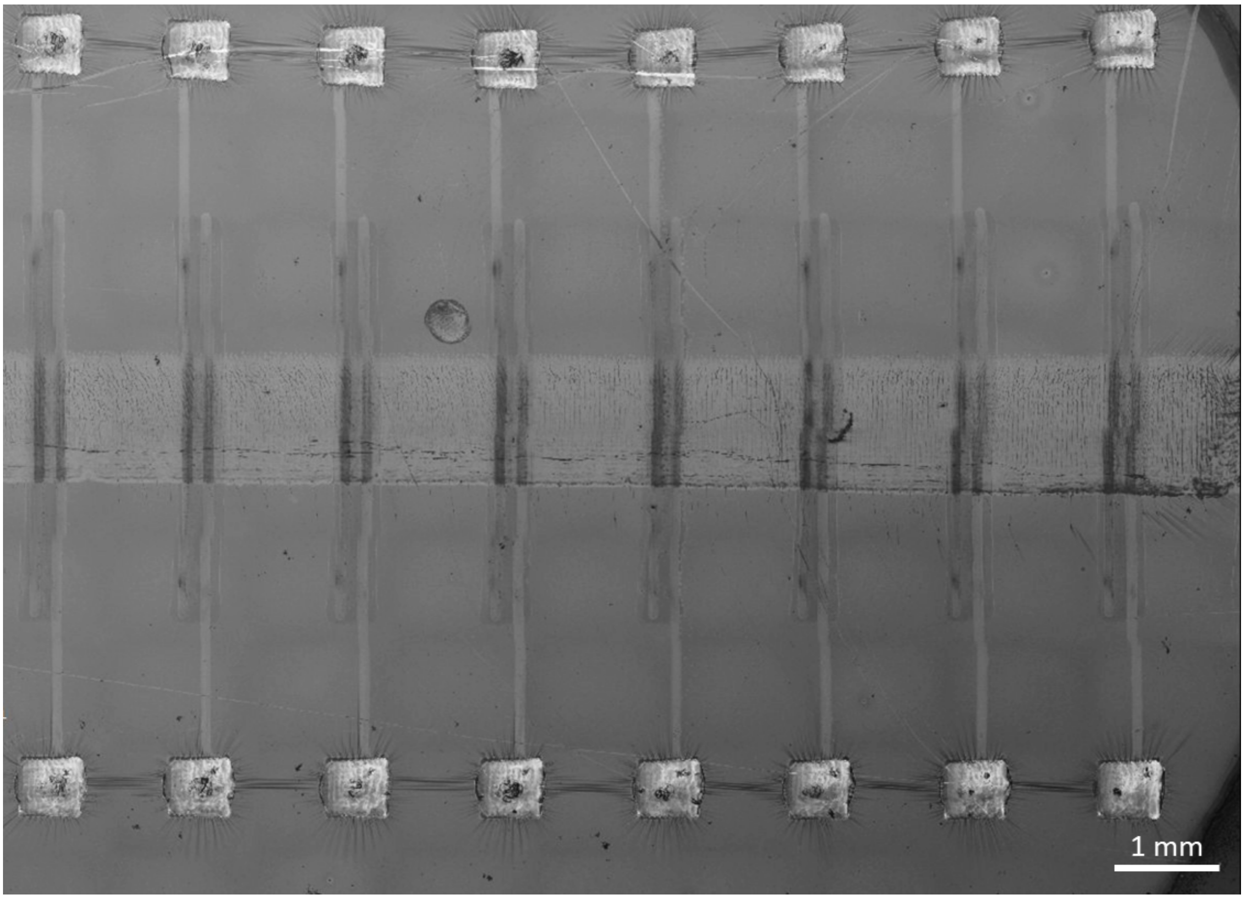


**Supplementary Fig. 1. Optical image of an array of 8 freestanding organic transistors.** Reference optical image of a typical freestanding film device obtained by Leica DCM 3D Confocal Profilometer, at 10x magnifications, with multiple images stitching option.

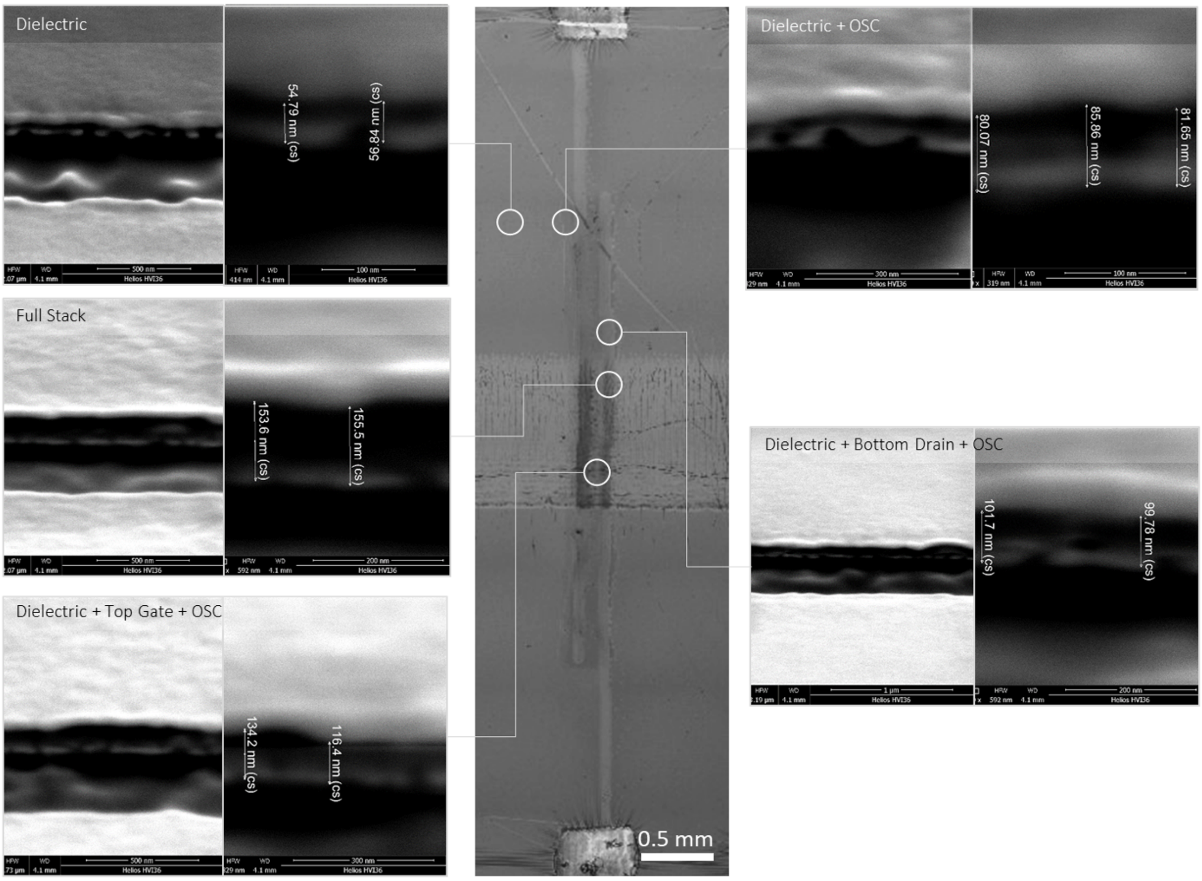


**Supplementary Fig. 2. Cross sections of the freestanding organic transistors.** Scanning Electron Microscopy image of cross section of the device in different positions obtained by Focus Ion Beam etching (sample tilt 52°, scale bar 200 nm, multiple magnification). Reference optical image obtained by Leica DCM 3D Confocal Profilometer, at 10x magnifications.

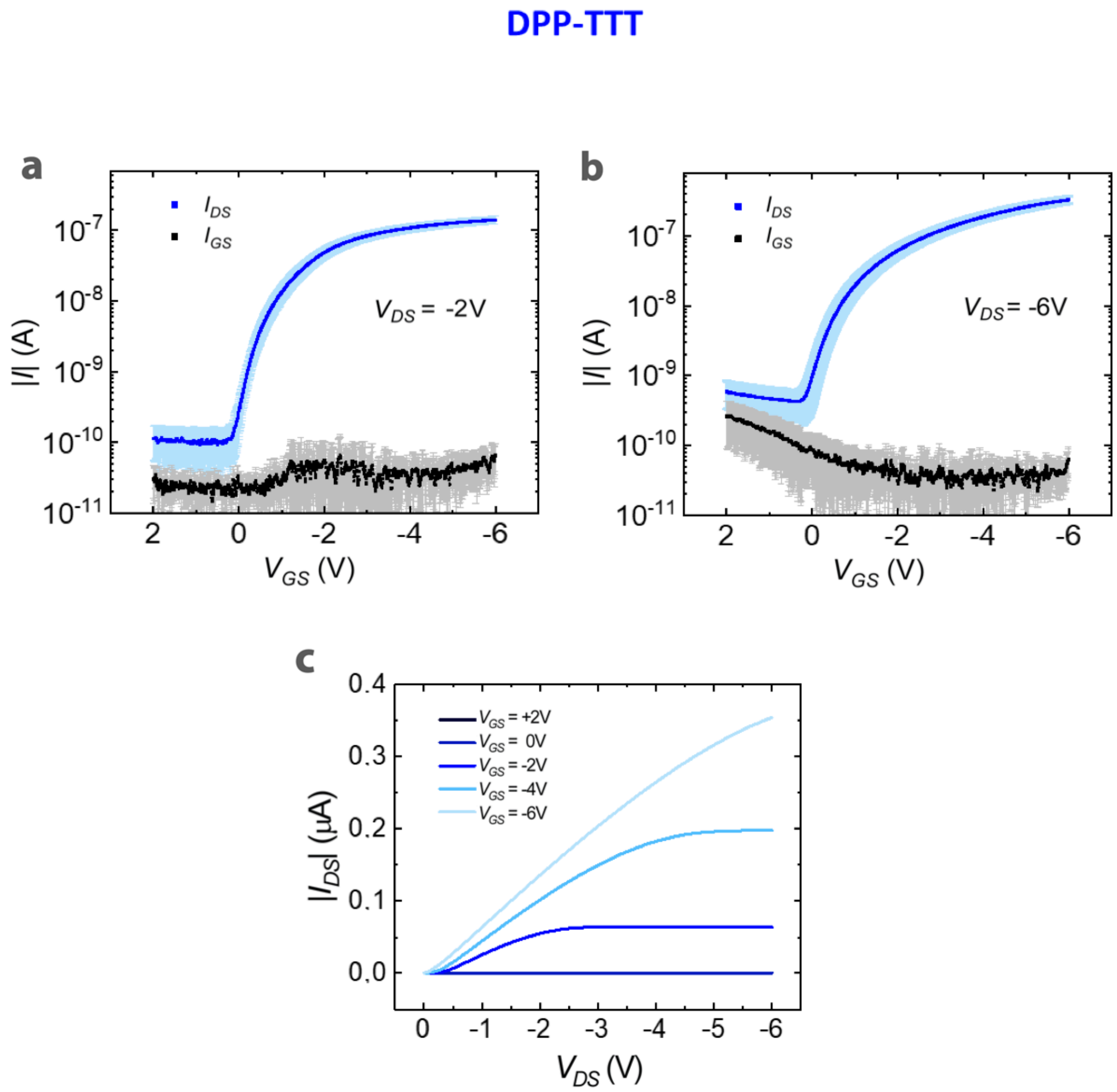


**Supplementary Fig. 3. Electrical characterization of DPP-TTT based organic transistors.** Statistic, with mean and standard deviation, of the transfer curves of 8 DPP-TTT based devices in **a)** linear and **b)** saturation mode; **c)** output curve for DPP-TTT based ultra-thin transistor.

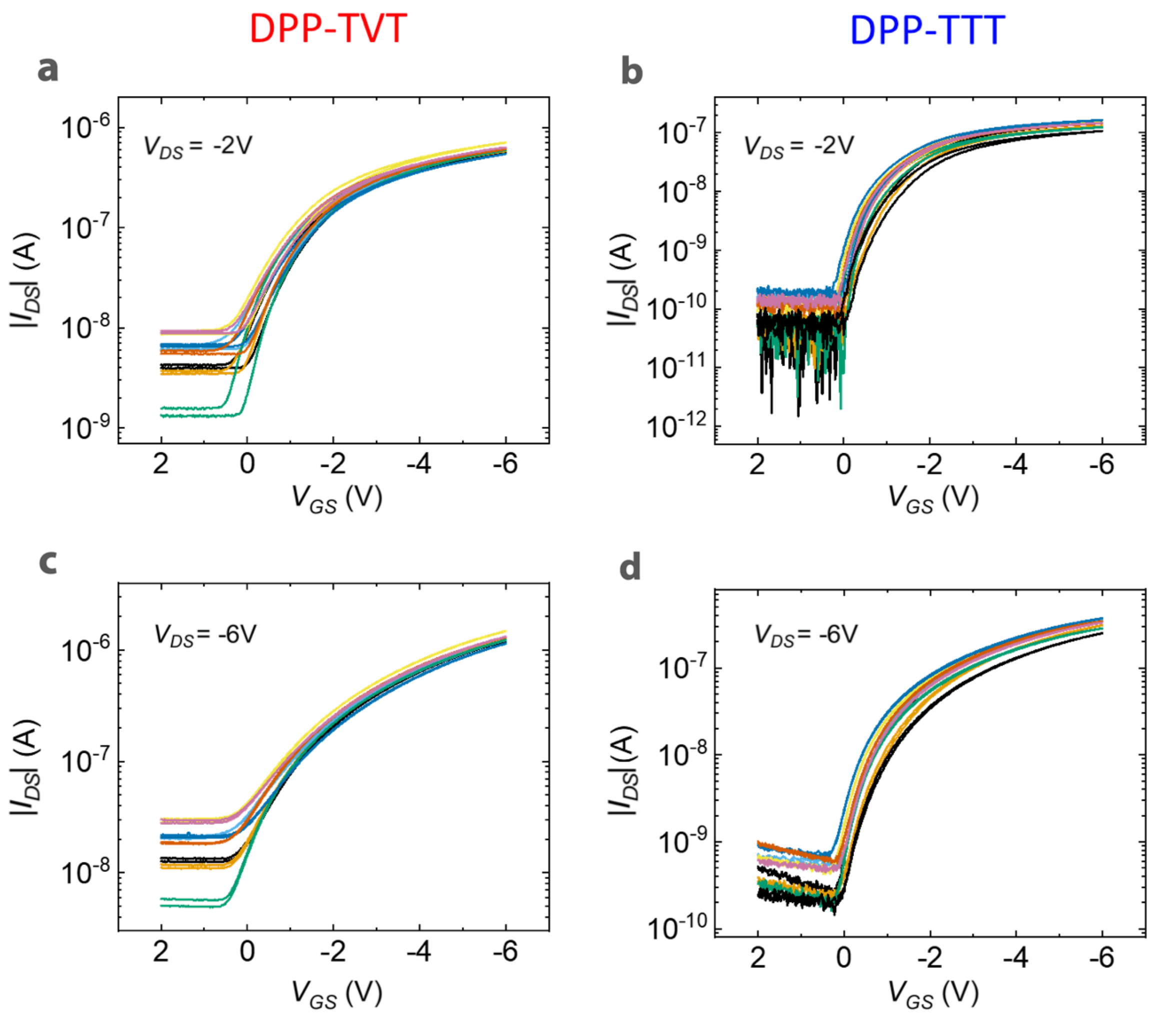


**Supplementary Fig. 4. Raw data of the electrical characterization of DPP-TVT and DPP-TTT based transistors.** Representative transfer curves for 29-DPP-TVT devices in **a)** linear and **c)** saturation mode, and for DPP-TTT devices in **b)** linear and **d)** saturation mode.

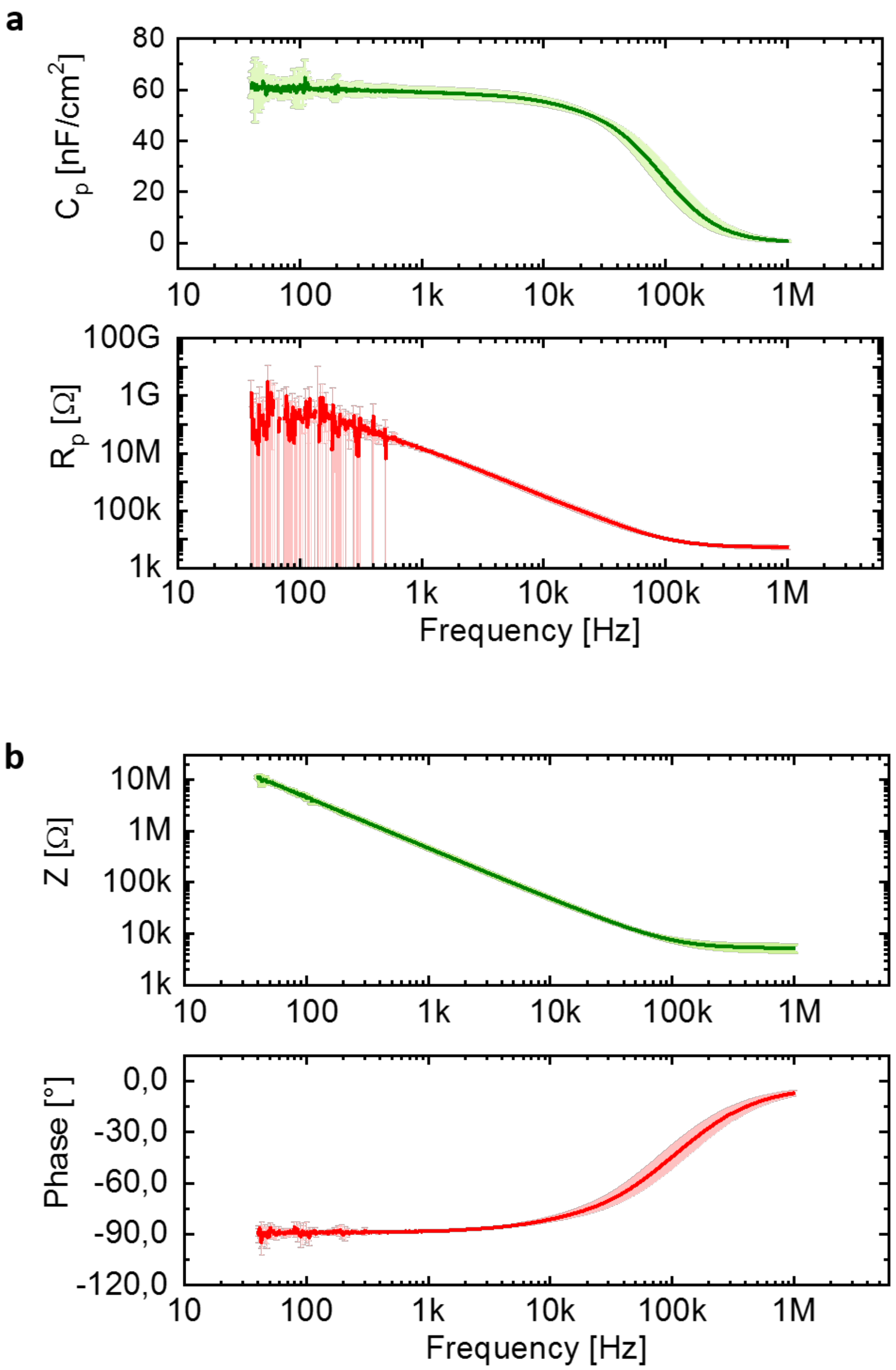


**Supplementary Fig. 5. PVF impedance measurements.** Statistics of 8 capacitors showing mean (full lines) and standard deviation (grey bars) for: **a)** $C_p$ (green) and $R_p$ (red) describing the impedance with a parallel equivalent circuit model; **b)** actual impedance module (green) and phase (red), as acquired.

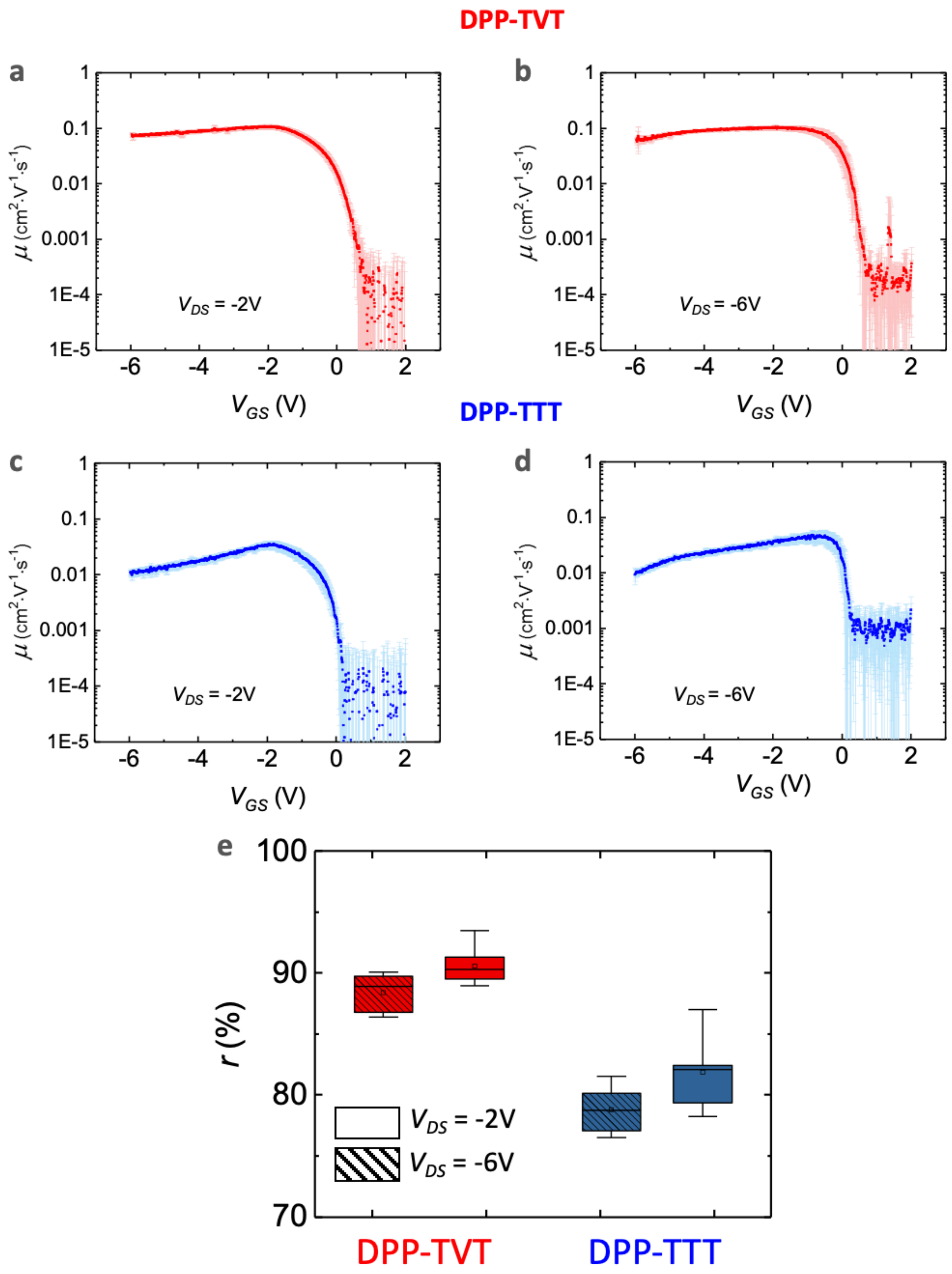


**Supplementary Fig. 6. Holes mobility values of 29-DPP-TVT and DPP-TTT devices.** Statistics, with mean and standard deviation, of the mobility values vs. gate voltage for the 29-DPP-TVT devices in **a)** linear and **b)** saturation mode, and for the DPP-TTT devices in **c)** linear and **d)** saturation mode; **e)** boxplot reporting the reliability factor r in linear and saturation regime for both 29-DPP-TVT and DPP-TTT based devices (square = mean, black line = median, each quartile group contains 25 % of the data).

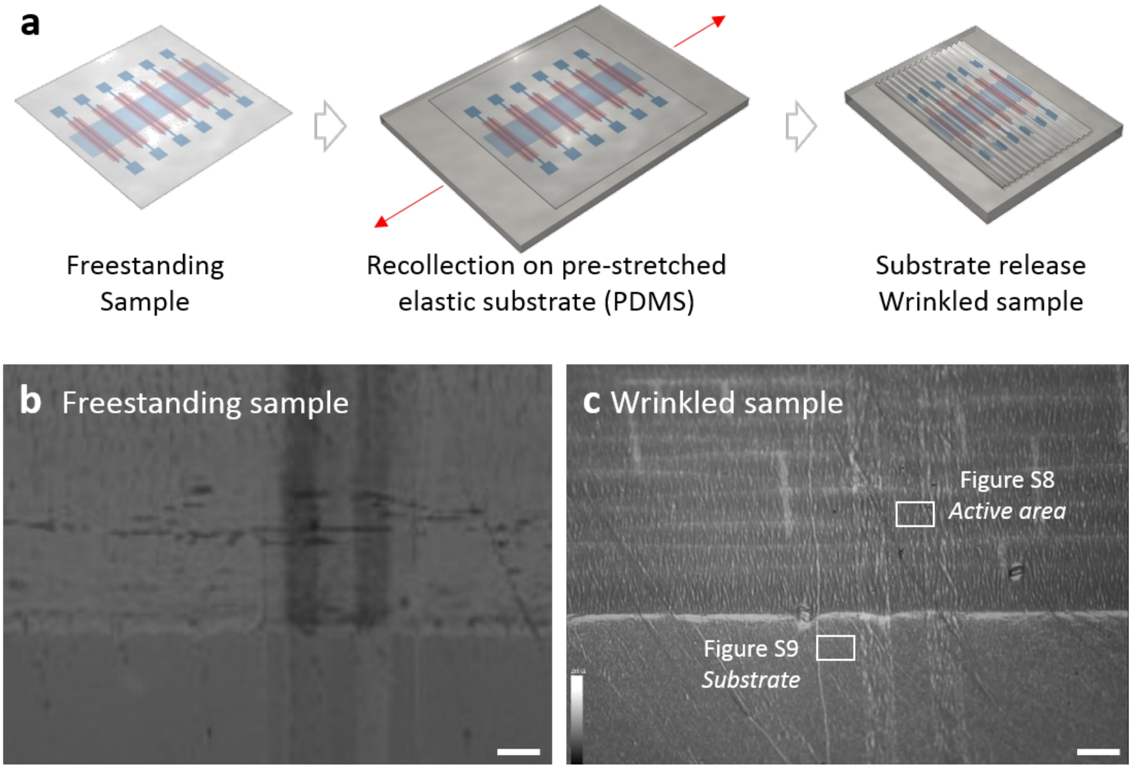


**Supplementary Fig. 7. Freestanding and wrinkled transistors. a)** Procedure to induce wrinkling and high curvature in the sample. The freestanding sample is transferred (recollected) on a pre-stretched elastic substrate (in our case polydimethylsiloxane (PDMS), pre-stretching 5%) and electrically characterized (flat sample). After that the pre-stretched PDMS is released and the sample stitched on top of substrate (that has higher Young's modules respect to the substrate) forms bucking and wrinkles, as in strain-induced elastic buckling instability for mechanical measurements (SIEBIMM) technique [Stafford CM et al., Nat Mater. 2004;3(8):545-550]. The device is then electrically characterized in wrinkle state, to demonstrate that high degree of curvature does not impair the functionality; **b)** optical picture of a freestanding device; **c)** the same area of **b)** after recollection and wrinkling; the white boxes indicate the points in which the specific curvature analysis is performed, as reported in Supplementary Fig. 8 (active area) and Supplementary Fig. 9 (substrate area). Scale bars 100 μm.

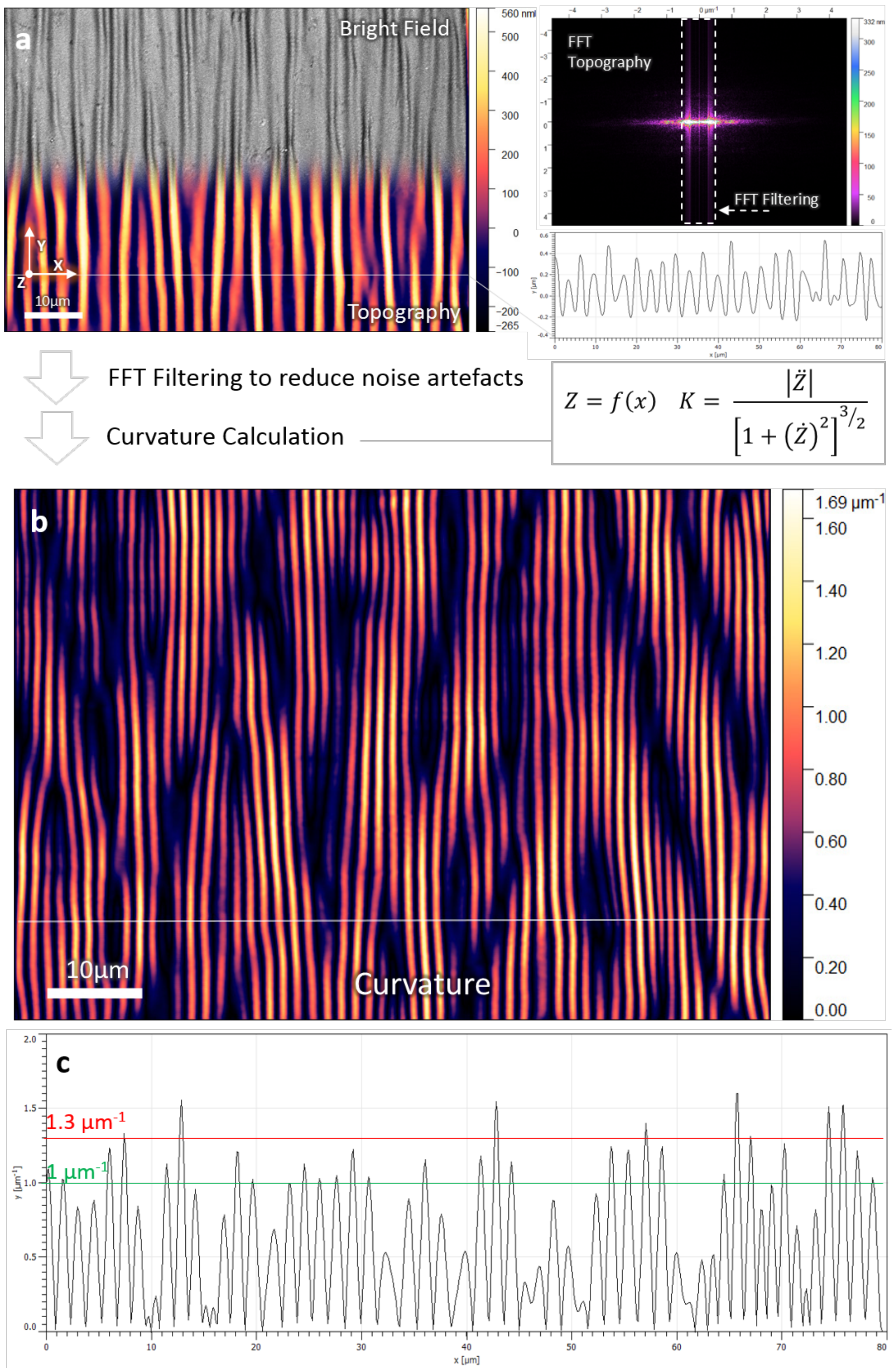


**Supplementary Fig. 8. Curvature analysis over the transistor channel.** Curvature mapping of wrinkled sample in active area region (as specified in Supplementary Fig. 7). **a)** Merged optical and topographical images acquired Leica DCM 3D Confocal Profilometer, at 150x magnifications. In the right insets a FFT image of the topography map of the same region and below a typical profile curve. The topography map has been elaborated by means of Gwydion

software by applying a FFT filter (to reduce noise artefacts) and subsequently calculating the curvature of the surface (K) along the x axis (as reported in figure), thus obtaining the curvature mapping, as reported in **b)**. **c)** Typical curvature profile for the considered area: more than 50% of the curvature maxima show $K > 1\ \mu m^{-1}$ (curvature radius $< 1\ \mu m$), more than 10% of the curvature maxima show $K > 1.3\ \mu m^{-1}$ (curvature radius $< 0.8\ \mu m$).

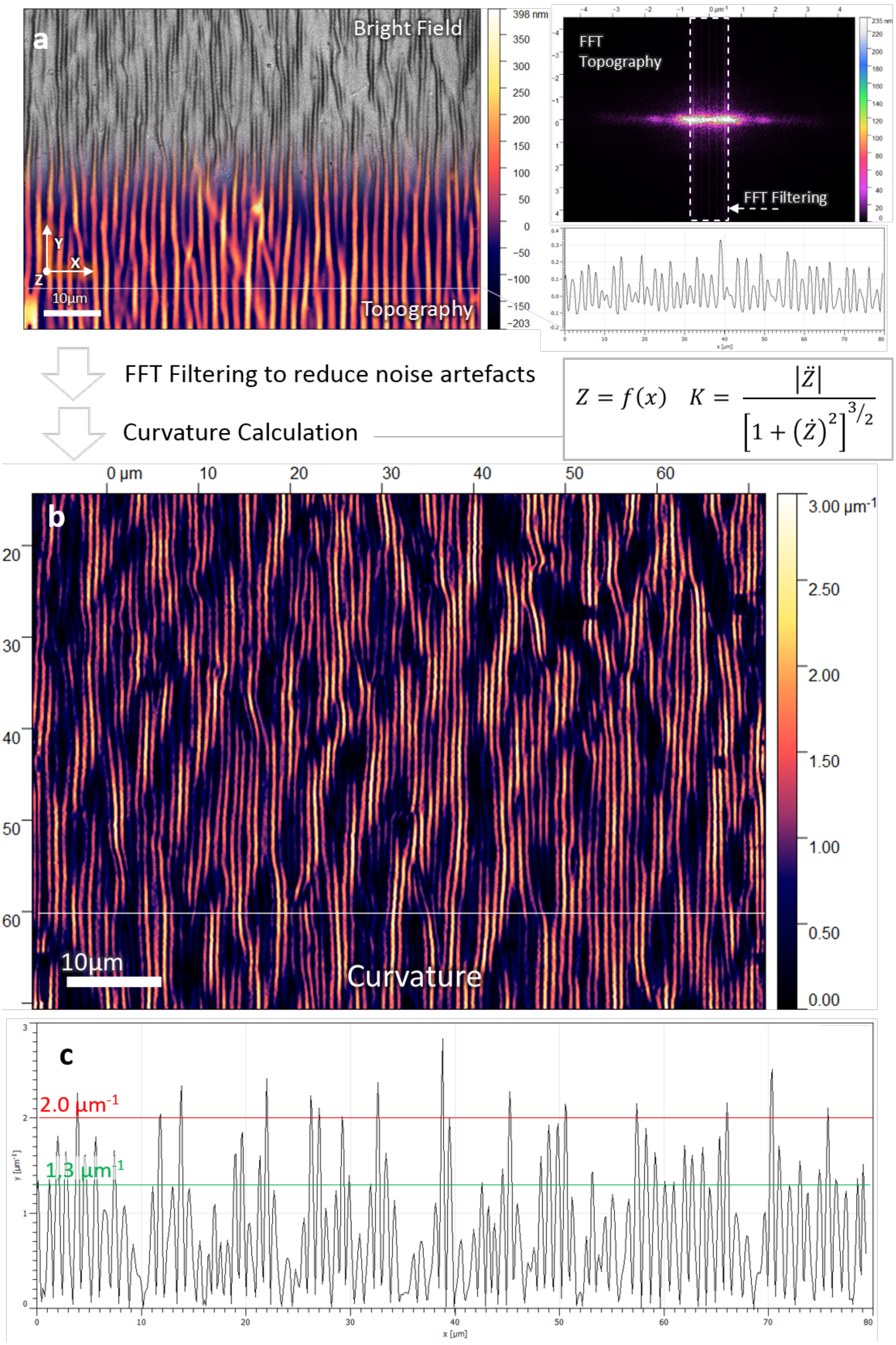


**Supplementary Fig. 9. Curvature analysis over PVF substrate**. Curvature mapping of wrinkled sample in substrate area region (as specified in Supplementary Fig. 7). **a)** Merged optical and topographical images acquired Leica DCM 3D Confocal Profilometer, at 150x magnifications. In the right insets a FFT image of the topography map of the same region and below a typical profile curve. The topography map has been elaborated by means of Gwydion

software by applying a FFT filter (to reduce noise artefacts) and subsequently calculating the curvature of the surface (K) along the x axis (as reported in figure), thus obtaining the curvature mapping, as reported in **(b)**. **c)** Typical curvature profile for the considered area: more than 50% of the curvature maxima show K > 1.3 $\mu m^{-1}$ (curvature radius < 0.8 μm), more than 10% of the curvature maxima show K > 2 $\mu m^{-1}$ (curvature radius < 0.5 μm).

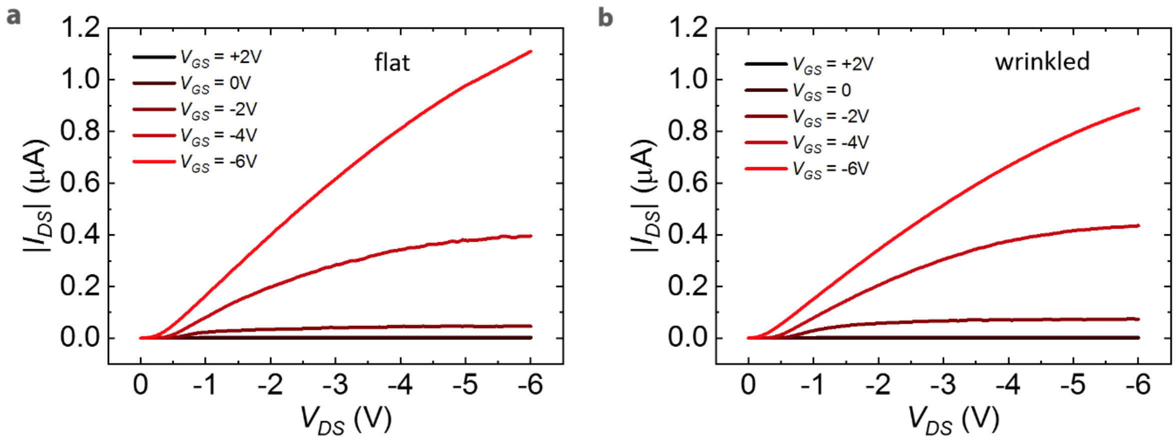


**Supplementary Fig. 10. Mechanical flexibility and conformability. a)** Output curve for a ultra-thin device when recollected on a pre-stretched PDMS substrate (flat sample) and **b)** after PMDS relaxation, which induces a strong buckling on the surface (wrinkled sample).

To assess the reproducibility run-to-run, another array of 8 devices was fabricated and characterized (Batch 2), by employing as organic semiconductor 29-DPP-TVT, since it showed higher electrical performance with respect to DPP-TTT (see Batch 1 – Supplementary Fig. 6). From the transfer characteristics in linear and saturation regime (Supplementary Fig. 11), it is possible to appreciate the low voltage operation of the devices, with an average Off and On currents slightly lower compared to those of the Batch 1 (reported in Fig. 3a-b) and same mobility (Supplementary Fig. 12c). We attribute these slightly different values to a lower p-type doping of the organic semiconductor, which is a well-known effect in 29-DPP-TVT based transistors (as extensively reported in in J. H. Lee et al. *RSC Adv.* **13**, 2017). The $I_{GS}$ gate leakage currents values are below 3 nA in saturation, more than 3 orders of magnitude lower than the On currents.

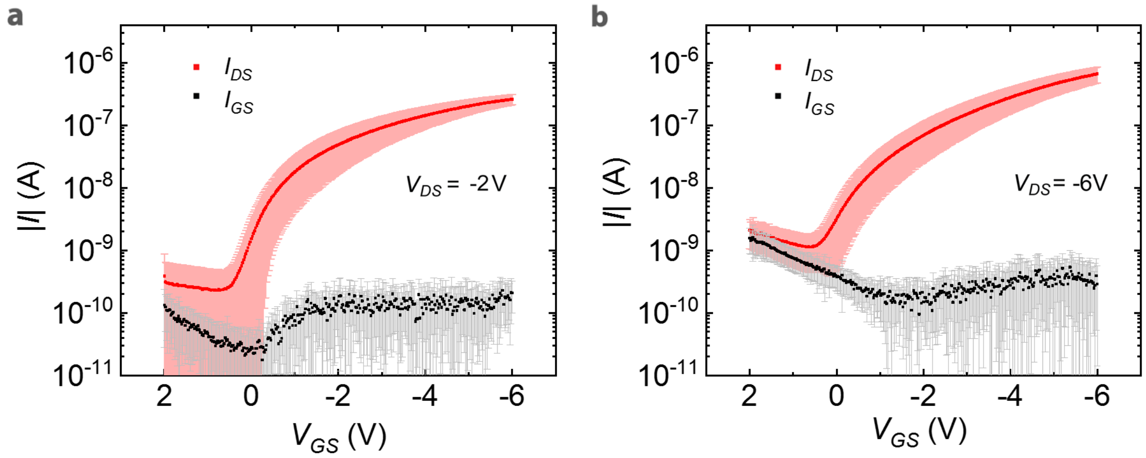


**Supplementary Fig. 11. Electrical characterization of the organic transistors.** Average transfer characteristic curves, with their standard deviation, obtained on 8 ultra-thin devices (Batch 2) based on 29-DPP-TVT in **a)** linear and **b)** saturation regime.

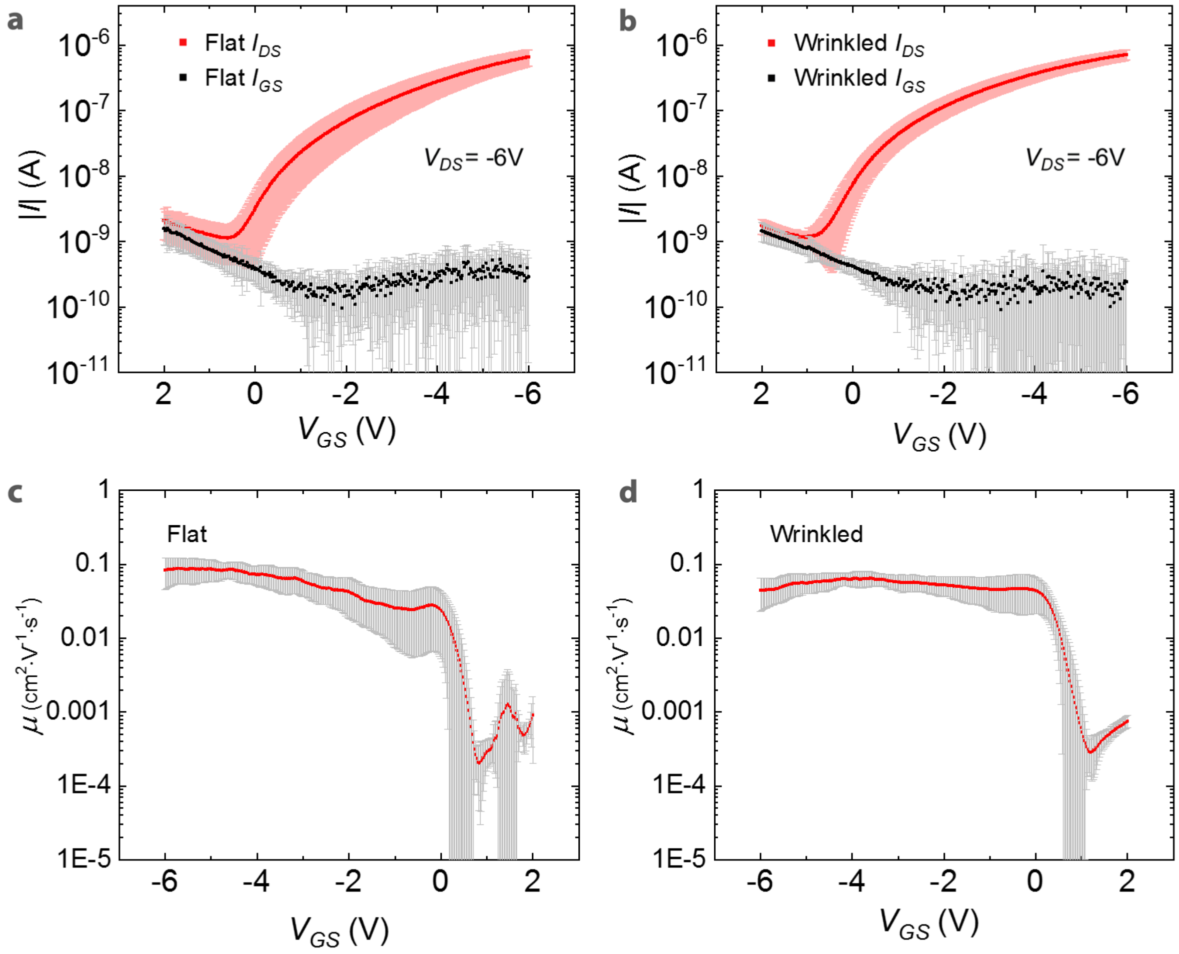


**Supplementary Fig. 12. Statistics of mechanical flexibility and conformability. a)** Average transfer characteristic curves, with their standard deviation, obtained on 8 ultra-thin devices (Batch 2) based on 29-DPP-TVT in saturation mode when recollected on a pre-stretched PDMS substrate (flat sample) and **b)** after PMDS relaxation, which induces a strong buckling on the surface (wrinkled sample). **c)** Statistics, with mean and standard deviation, of the mobility values vs. gate voltage for the 29-DPP-TVT devices (Batch 2) when recollected on a pre-stretched PDMS substrate (flat sample) and **d)** after PMDS relaxation, which induces a strong buckling on the surface (wrinkled sample).

**Supplementary Table 1.** Average Effective Mobility.

| | DPP-TVT | | DPP-TTT | |
|---|---|---|---|---|
| | **Saturation** | **Linear** | **Saturation** | **Linear** |
| Average $\mu_{eff}$ ($cm^{-2} \cdot V^{-1} \cdot s^{-1}$) | 0.087 | 0.083 | 0.027 | 0.020 |
| SD ($cm^{-2} \cdot V^{-1} \cdot s^{-1}$) | 0.006 | 0.007 | 0.003 | 0.003 |

**Supplementary Table 2.** Average *r* Values.

| | DPP-TVT | | DPP-TTT | |
|---|---|---|---|---|
| | **Saturation** | **Linear** | **Saturation** | **Linear** |
| Average *r* (%) | 0.884 | 0.906 | 0.788 | 0.819 |
| SD (%) | 0.014 | 0.014 | 0.017 | 0.027 |

**Supplementary Table 3.** Average Apparent Mobility Values.

| | DPP-TVT | | DPP-TTT | |
|---|---|---|---|---|
| | **Saturation** | **Linear** | **Saturation** | **Linear** |
| Average $\mu$ ($cm^{-2} \cdot V^{-1} \cdot s^{-1}$) | 0.098 | 0.092 | 0.035 | 0.025 |
| SD ($cm^{-2} \cdot V^{-1} \cdot s^{-1}$) | 0.007 | 0.007 | 0.004 | 0.003 |

**Supplementary Table 4.** Contact resistance Values.

| | DPP-TVT | DPP-TTT |
|---|---|---|
| Average $R_C \times W$ (kΩ·cm) | 85 | 769 |
| SD (kΩ·cm) | 19 | 109 |

The values of the contact resistance were extracted from the transfer curves in linear regime (@$V_{DS}$ = -2 V) using the Y-function method reported in Y. Xu et al. (J. Appl. Phys. 2010, 107 - DOI: 10.1063/1.3432716). According with Kim (Appl. Phys. Rev. 2020, 7, 031306 - DOI: 10.1063/5.0005441) it is possible to decoupling the effect of the contact resistance ($R_C$) from the channel resistance ($R_{ch}$) and roughly estimate the holes mobility ($\mu_{real}$). In our devices, the

extracted value of $\mu_{real}$ is ≅ 0.2 $cm^2 V^{-1} s^{-1}$ for the 29-DPP-TVT based devices and 0.08 $cm^2 V^{-1} s^{-1}$ for the DPP-TTT based devices.

**Supplementary Table 5.** Sub-threshold swing Values.

| DPP-TVT based devices | **Sub-threshold swing (V/decade)** | |
|---|---|---|
| | **mean** | **standard deviation** |
| Batch 1 (flat) | 1.8 | 0.4 |
| Batch 2 (flat) | 0.9 | 0.3 |
| Batch 2 (wrinkled) | 1.0 | 0.1 |

**Supplementary Table 6.** Threshold voltage Values.

| DPP-TVT based devices | **Threshold voltage (V)** | |
|---|---|---|
| | **mean** | **standard deviation** |
| Batch 1 (flat) | 0.9 | 0.2 |
| Batch 2 (flat) | 0.4 | 0.2 |
| Batch 2 (wrinkled) | 0.7 | 0.2 |

**Supplementary Table 7.** Summary and comparison of super-thin and ultra-thin organic field effect transistors, with total thickness and minimum bending radius sustainable.

| **Total thickness [nm]** | **Bending radius [µm]** | **Fabrication process** | | | | **Reference** |
|---|---|---|---|---|---|---|
| > 4000 | n. a. | ***OSC*** | ***Electrodes*** | ***Dielectric*** | ***Substrate*** | 22 |
| | | Printed | Printed | CVD | CVD | |
| 1000-3000 | n. a. | ***OSC*** | ***Electrodes*** | ***Dielectric*** | ***Substrate*** | 24 |
| | | Printed | Printed | CVD | CVD | |
| 2000 | 140 | ***OSC*** | ***Electrodes*** | ***Dielectric*** | ***Substrate*** | 23 |
| | | Printed | Printed | Spin coating | CVD + Spin coating | |
| 1200 | 200 | ***OSC*** | ***Electrodes*** | ***Dielectric*** | ***Substrate*** | 21 |

| | | | | | | |
|---|---|---|---|---|---|---|
| | | Drop casting | Thermal evaporation | CVD | CVD | |
| 630 | 1.5 | ***OSC*** | ***Electrodes*** | ***Dielectric*** | ***Substrate*** | 31 |
| | | Thermal evaporation | Thermal evaporation | Anodization (Alumina) | Spin coating | |
| 600 | n. a. | ***OSC*** | ***Electrodes*** | ***Dielectric*** | ***Substrate*** | 20 |
| | | Drop casting / thermal evaporation | Thermal evaporation | CVD | CVD | |
| 590 | 6500 | ***OSC*** | ***Electrodes*** | ***Dielectric*** | ***Substrate*** | 29 |
| | | Thermal evaporation | Thermal evaporation | Spin coating | Spin coating | |
| 550 | 2000 | ***OSC*** | ***Electrodes*** | ***Dielectric*** | ***Substrate*** | 19 |
| | | Thermal evaporation | Thermal evaporation | CVD | CVD | |
| 480 | n. a. | ***OSC*** | ***Electrodes*** | ***Dielectric*** | ***Substrate*** | 28 |
| | | Drop casting | Thermal evaporation | Spin coating | Spin coating | |
| 380 | 280 | ***OSC*** | ***Electrodes*** | ***Dielectric*** | ***Substrate*** | 30 |
| | | Spin coating | Thermal evaporation | Spin coating | Spin coating | |
| 350 | 2 | ***OSC*** | ***Electrodes*** | ***Dielectric*** | ***Substrate*** | 25 |
| | | Thermal evaporation | Thermal evaporation | CVD | CVD | |
| 320 | 5 | ***OSC*** | ***Electrodes*** | ***Dielectric*** | ***Substrate*** | 32 |
| | | Thermal evaporation / Spin coating | Thermal evaporation | Spin coating | Spin coating | |
| 320 | 6500 | ***OSC*** | ***Electrodes*** | ***Dielectric*** | ***Substrate*** | 27 |
| | | Spin coating | Thermal evaporation | Spin coating | Spin coating | |
| 270 | 1.5 | ***OSC*** | ***Electrodes*** | ***Dielectric*** | ***Substrate*** | 26 |
| | | Thermal evaporation | Thermal evaporation | CVD | CVD | |
| 143 | 0.7 | ***OSC*** | ***Electrodes*** | ***Dielectric*** | ***Substrate*** | This work |
| | | Printed | Printed | Spin coating | Spin coating | |
