# Supplementary material for: A sub-150-nanometre-thick and ultraconformable solution-processed all-organic transistor
Source: Nat Commun. 2021 Oct 6;12:5842. doi: 10.1038/s41467-021-26120-2 (PMC8494881; doi:10.1038/s41467-021-26120-2)
Supplement: Supplementary file 2 — Description of Additional Supplementary Files [file 41467_2021_26120_MOESM2_ESM.pdf]

Description of Additional Supplementary Files

File Name: Supplementary Movie 1
Description: Ultrathin OFETs fabrication – delamination and recollection of the PVF nanosheet.
